# Assessment of the anti-nociceptive effects of fetal ventral mesencephalic tissue allografts in a rat model of hemi-Parkinson’s disease using fMRI

**DOI:** 10.3389/fnagi.2022.948848

**Published:** 2022-11-18

**Authors:** Chuang-Hsin Chiu, Shao-Ju Weng, Skye Hsin-Hsien Yeh, Yun-Ting Jhao, Hsien-Feng Chang, Wen-Sheng Huang, Cheng-Yi Cheng, Chun-Chang Yeh, Kuo-Hsing Ma

**Affiliations:** ^1^Department of Nuclear Medicine, National Defense Medical Center, Tri-Service General Hospital, Taipei, Taiwan; ^2^Department of Biology and Anatomy, National Defense Medical Center, Taipei, Taiwan; ^3^School of Medicine, National Defense Medical Center, Taipei, Taiwan; ^4^Yuan Rung Hospital, Changhua, Taiwan; ^5^Department of Nuclear Medicine, Cheng-Hsin General Hospital, Taipei, Taiwan; ^6^Department of Anesthesiology, National Defense Medical Center, Tri-Service General Hospital, Taipei, Taiwan

**Keywords:** Parkinson’s disease, motor, non-motor, fMRI, neuroimaging, fetal ventral mesencephalic tissue

## Abstract

Extensive studies showed increased subjective pain sensitivity in Parkinson’s disease (PD), which appeared to be partially reversed by dopaminergic (DA) treatment. Although cell replacement represents an attractive therapeutic strategy, its potential for PD-related hyperalgesia remains unclear. We investigated re-establishment of DA function *via* allografting exogenic DA cells on pain hypersensitivity in a rat model of PD. We evaluated the anti-nociceptive effects of fetal ventral mesencephalic (rVM) tissue allografts in PD rats after unilateral 6-OHDA-induced toxicity in the medial forebrain bundle. The drug –induced rotation test was used to validate the severity of the nigrostriatal lesion; von Frey and thermal pain tests were employed to evaluate nociceptive function. Nociception-induced cerebral blood volume (CBV) response was measured using a 4.7-T MR system. Finally, the immunohistochemical (IHC) studies were performed and the results were compared with the imaging findings from functional magnetic resonance imaging (fMRI). The grafts significantly improved drug-induced rotation behavior and increased mechanical and thermal nociceptive thresholds in PD rats. The elevation of CBV signals significantly recovered on the grafted striatum, whereas this effect was inhibited by the D2R antagonist eticlopride in each striatum. Quantitative IHC analysis revealed the transplantation markedly increased the numbers of tyrosine hydroxylase immunoreactive cells. Therefore, we concluded transplantation of rVM tissue results in anti-nociceptive effects and improves motor function. Moreover, *in vivo* CBV response confirmed the key role of D2R-mediated pain modulation. Therefore, we demonstrate fMRI as a reliable imaging index in evaluating the anti-nociceptive therapeutic effects of fetal rVM transplantation in the rat model of PD.

## Introduction

Parkinson’s disease (PD) is the second most common neurodegenerative disorder after Alzheimer’s disease, and the incidence and prevalence of patients with PD increase steeply with age (Huse et al., 2005). The symptoms of patients with PD may include physical or motor symptoms such as tremors, slowing, stiffening movement, and balance problems (Wasner and Deuschl, 2012). In addition to the motor dysfunction, studies have shown nonmotor symptoms (NMS), such as depression, psychosis, pain, and sleep disturbances, as the important factor for the quality of life of patients with PD (Wasner and Deuschl, 2012). Pain is the most frequent NMS at the onset of disease, and its prevalence increases during the progression of PD (Lee et al., 2006). Pain may precede the onset of motor parkinsonian symptoms and be related to motor fluctuations, early morning dystonia, or secondary causes such as musculoskeletal pain (Quinn et al., 1996). The prevalence of all types of pain is high, but variable. The prevalence of pain is estimated to be between 40 and 85% in patients with PD, with these variations likely due to differences in the study designs, pain assessment methods, or definitions of pain (Cuomo et al., 2019). PD-associated pain is not only a clinically relevant symptom (Djaldetti et al., 2004; Beiske et al., 2009), but is also often overlooked as a consequence of the changes in motor function.

The nigrostriatal pathway has been reported to respond to nociceptive stimuli and also exerts antinociceptive activity (Brooks, 2006). Lesioned nigral dopaminergic (DA) neurons have been proven to exhibit increased sensitivity to pain (Lin et al., 1981; Dieb et al., 2014), whereas activation of DA neurons inhibits the response to pain (Chudler and Dong, 1995). Moreover, clinical evidence and experimental studies have demonstrated the involvement of basal ganglia circuitry in pain modulation. For example, somatosensory information is processed by the basal ganglia *via* several mechanisms, and abnormalities in the basal ganglia affect the encoding of pain (Djaldetti et al., 2004). Moreover, endogenous pain in patients with PD is frequently associated with a higher sensitivity to painful stimuli, and many patients with PD have lower pain thresholds for electrical and heat stimuli (Mylius et al., 2009). Patients at an early stage of the disease also tend to have increased responses to nociceptive stimuli and lower pain thresholds such as enhanced spinal nociceptive reflexes (Zambito Marsala et al., 2011), whereas pain sensory discrimination, e.g., subjective estimation of provoked pain, remains unaltered (Mylius et al., 2009). Enhanced sensitivity to nociceptive stimuli was previously attributed to functional changes at the spinal level (Braak et al., 2007) and within the pain matrix (Gerdelat-Mas et al., 2007), a set of brain areas including the medial pain pathway mainly including the thalamus, the primary and secondary somatosensory cortices (S1 and S2), the anterior/mid cingulate cortex (ACC/MCC) and the insula that consistently respond to painful, as indicated by PET (Brefel-Courbon et al., 2005; Gerdelat-Mas et al., 2007; Mylius et al., 2009). These changes were reversed by dopaminergic treatment, indicating a major role for dopamine depletion in PD-associated pain (Gerdelat-Mas et al., 2007). Taken together, this evidence indicates that numerous circuits at multiple CNS levels are involved in the pain in PD, largely due to anatomical projections of the basal ganglia, which connect specific striatal areas, i.e., the putamen, caudate, and nucleus accumbens (Alexander, 2004).

In the past decade, researchers have undertaken efforts to investigative the mechanisms that underlie the pathology of PD-related pain. In rodents, both systemic administration of the neurotoxin 1-methyl-4-phenyl-1,2,3,6-tetrahydropyridine (MPTP) and intracranial injection of 6-hydroxydopamine (6-OHDA) induce severe lesions of DA neurons in the substantia nigra pars compacta (SNc), and mimic the characteristic pathophysiology of PD. Reduced mechanical and thermal nociceptive thresholds in the hindpaw were demonstrated in these animal models, and this hypersensitivity can be relieved by systemic administration of dopamine receptor agonists (Park et al., 2015; Domenici et al., 2019). Tang et al. (2021) reported that pain hypersensitivity in PD mice was associated with hyperexcitability of superficial dorsal horn neurons, and both were reversed by activation of spinal D2 receptors (Tang et al., 2021). Powerful imaging techniques such as functional magnetic resonance imaging (fMRI) enabled *in vivo* visualization of decreased cerebral blood volume (CBV) in response to nociceptive stimuli in the striatum under normal conditions *via* DA neurotransmission (Chang and Shyu, 2001; Liu et al., 2004; Zhao et al., 2008; Shih et al., 2009). Furthermore, nociception-induced changes in the striatal CBV signals with specificity for DA dysfunction were observed in a rat model of PD, and may be characterized as the pain symptoms of PD (Chen et al., 2013).

Treatment of pain depends on the type of pain and usually requires a multidisciplinary approach. Studies have shown that levodopa (L-dopa) can increase the pain threshold in patients with PD (Brefel-Courbon et al., 2005; Gerdelat-Mas et al., 2007; Schestatsky et al., 2007). Dopamine receptor agonists have also been demonstrated to relieve nociceptive pain, as described above. Thus, these data suggest that dopamine depletion could contribute to changes in nociceptive processing in PD-related pain, and that dysfunction of the nigrostriatal dopamine system may directly or indirectly affect pain signaling at the spinal cord level. However, the mechanisms how dopamine and its receptors in striatum and spinal cord contribute to pain hypersensitivity in PD remain largely unknown.

Cell therapy in PD have been a major area of research for the last 30 years, with the main focus on using cells to replace the degenerating and lost DA neuronal innervation of the striatum from the nigra (Wijeyekoon and Barker, 2009; Fan et al., 2020). Much experience and some success have been gained from fetal ventral mesencephalic tissue transplants, thus the rapidly advancing field of stem cells may provide attractive alternative options for the treatment of PD (Kordower et al., 1995; Guo et al., 2021). Numerous animal studies have shown that transplantation of DA neurons using either rat ventral mesencephalic (rVM) neural tissue (Weng et al., 2017, 2020), human embryonic stem cells or human fetal ventral mesencephalic neural tissue (Rath et al., 2013; Grealish et al., 2014) improves the motor symptoms in rodent models of PD. However, to our knowledge, no report has validated the potential therapeutic effect of rVM cell transplantation on PD-related nociceptive pain.

One of the major research interests of our group over the past decade has been to investigate the therapeutic effects of transplantation of rVM neural tissue using *in vivo* molecular imaging, i.e., positron emission tomography (PET) using 4-[^18^F]-ADAM targeted serotonin transport or [^18^F] DOPA targeted dopamine synthesis, in 6-OHDA-induced animal PD model (Weng et al., 2013; Chiu et al., 2016; Jhao et al., 2019). Based on the research experience described above, in this study, we hypothesized that transplantation of fetal rVM would beneficially improve the recovery of DA dysfunction in nociceptive impairment. Thus, the aim of this study was to evaluate the feasibility of fMRI as an *in vivo* imaging index for the therapeutic effects of fetal rVM transplantation on PD-related motor and non-motor dysfunction, i.e., pain, in a rat model of PD.

## Materials and methods

### Animals

All animal experiments were approved in advance by the institutional animal care and use committee (NDMC-15-207). Male Sprague Dawley (SD; age, 8-week-old) rats weighing 280–300 g (mean, 290 ± 10 g) from BioLASCO Taiwan Co., Ltd. (Taipei, Taiwan) were housed in the Animal Center of the National Defense Medical Center (Taipei, Taiwan), which is certified by the Association for Assessment and Accreditation of Laboratory Animal Care International (AAALAC International). The rats were housed at 23 ± 2°C under a controlled 12-h light/12-h dark cycle. All animals had free access to tap water and a complete pellet diet for 1 week before the experiments began. Only male animals were used in this study to avoid the influence from female reproductive cycles.

### 6-OHDA-induced rat hemi-parkinsonian model

**Figure 1:**
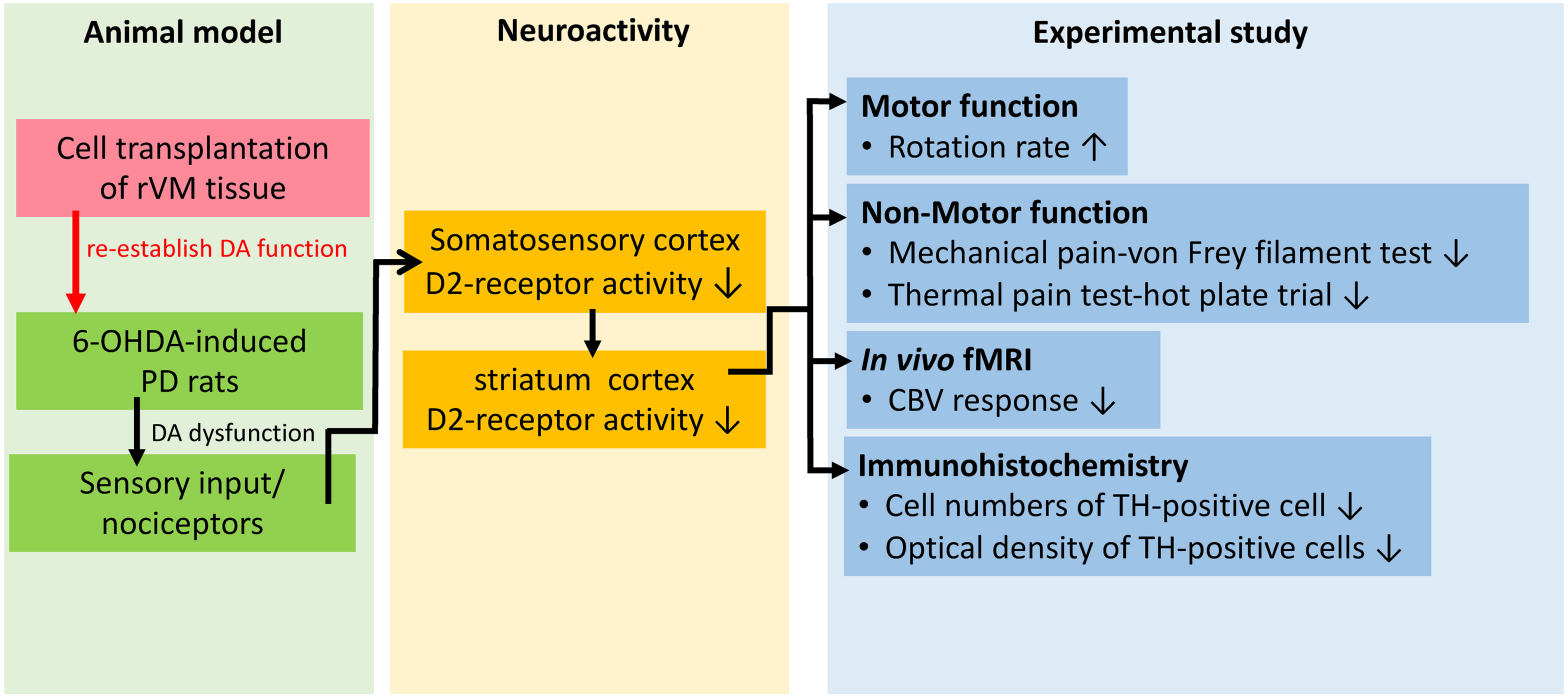
Conceptual framework of the study.

**Figure 2:**
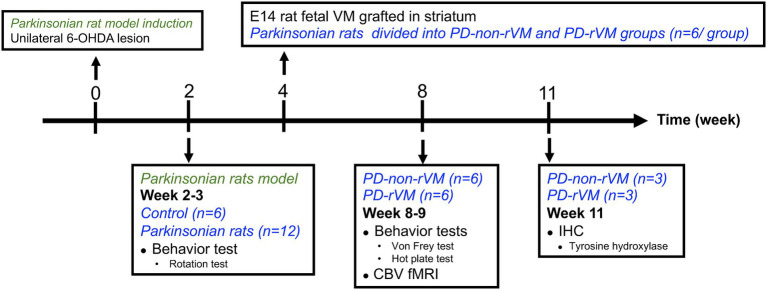
Schematic representation of the study design. Unilateral 6-OHDA lesions were induced at week 0 to establish the hemiparkinsonian model. Two weeks later, the animals were subjected to rotation tests; animals that met the PD criteria (>300  turns/h) were randomly allocated to either Group PD (without rVM treatment) or Group PD-rVM (with treatment). Group PD-rVM were transplanted with rVM tissue in week 4. All animals were subjected to behavior tests and CBV fMRI in week 8–9. Immunohistochemistry was performed in week 11.

The SD rats were used to establish the parkinsonian model (PD) by lesioning the right medial forebrain bundle (MFB) with 30 μg of 6-hydroxydopamine (6-OHDA; Sigma-Aldrich, Saint Louis, MO, United States) dissolved in 3 μl of ice-cold 0.02% ascorbic acid, as described previously (Weng et al., 2020). Briefly, after anesthetization by intraperitoneal injection of 0.4 ml/kg of 7% chloral hydrate (Riedel-de Haën, Seelze, Germany), the rats were placed in a stereotactic apparatus. The coordinates for the right MFB were 5.3 mm posteriorly, 2.1 mm laterally, and 8.2 mm ventrally from the bregma. The MFB/striatum was lesioned and the left MFB/striatum was used as the unlesioned side/self-control side. Figure 1 shows the conceptual framework of the study with the concept and Figure 2 presents a schematic representation of the study design.

The study described in the manuscript was conducted in the period 2011–2013; however, it should be noted that according to the guideline of the animal study protocol by the Institutional Animal Care and Use Committee guidelines at the National Defense Medical Center, Taipei, Taiwan, R.O.C., the use of chloral hydrate for anesthesia or euthanasia has been restricted in animal study since 2014.

### Motor behavior: Rotation test

Methamphetamine (METH) is an indirect agonist that induces the release of dopamine in the brain (Bustamante et al., 2002). Therefore, we assumed that the right MFB, i.e., the 6-OHDA-lesioned side, would have a much lower response to METH compared to the left, unlesioned MFB. After giving METH, the criteria for the PD model was defined as >300 turns/h (>5 turns/min; Bjorklund and Dunnett, 2019; Jhao et al., 2019). Two weeks after injection of 6-OHDA, 16 6-OHDA-induction and 6 sham control animals were used for METH-induced rotation. The experimental rats were intraperitoneally administered 2.0 mg/kg METH. The recording of rotational behavior was initiated 15 min post-injection and lasted for 60 min.

Based on the results of the rotation test, 12 parkinsonian rats (PD rats) were selected and randomly allocated into PD group: right-side 6-OHDA-lesioned and left-side unlesioned, without treatment (*n* = 6) or PD-rVM group: right-side 6-OHDA-lesioned and left-side unlesioned, and transplanted with rVM tissue (*n* = 6).

### Preparation and transplantation of rVM tissue

The PD-rVM group received transplantation/grafts. Briefly, rVM tissue dissected from embryonic E14 rat brains was grafted into the lesioned striatum of the PD-rVM group at week 4, as described by Dunnett, (2000) with some minor modifications (Weng et al., 2017). We ensured the presence of ventral mesencephalon and partial ventral pontine raphe in the dissected tissue slices, which were then placed in Hank’s Buffered Salt Solution (Gibco, Grand Island, NY, United States), dissected into small pieces, and transplanted into the ipsilateral striatum using glass microtubes (0.5 mm posterior to the bregma, 2.5 mm lateral to the midline, and 4.5 mm below the dura).

### Pain behavior: Von Frey and hot plate tests

#### Mechanical pain: Von Frey filament test

The threshold of mechanical pain was measured in acclimated rodents *via* the up-down method using von Frey filaments on the plantar surface. Briefly, the rats were placed individually in small cages with a mesh for the von Frey test, and a monofilament (Bio-VF-M; Bioseb, Vitrolles, France) was applied perpendicular to the plantar surface of the hindpaw until the paw buckled. A score ranging between 1.5 and 90 g was assigned in four consecutive positive responses to filaments with decreasing force or three consecutive negative responses to filaments with increasing forces. Nociceptive behaviors, including brisk paw withdrawal, licking, or shaking of the paw, in two trials were considered a positive response. The 50% paw withdrawal threshold (PWT) was determined as described previously (Zhou and Luo, 2015).

#### Thermal pain test: Hot plate trial

The hot plate trial was performed 1 day after the von Frey test. Rats were acclimatized for at least 30 min in the test compartment. A heat source (55°C) was positioned on the plantar surface of the hindpaw. Three hot plate trials were conducted, and the mean paw withdrawal latency was recorded. A 20 s cutoff time was used to prevent tissue damage.

#### Brain imaging: CBV fMRI

At weeks 8 ~ 9, the rats in the PD and PD-rVM groups (*n* = 6/group) were anesthetized by intravenous injection of 70.0 mg/kg α-chloralose (dissolved in pre-warmed 0.9% saline and 10% polyethylene glycol) for fMRI on a 4.7-T spectrometer (Biospec 47/40; Bruker, Bremen, Germany). A 72 mm volume coil was employed as the radio-frequency transmitter, with a quadrature surface coil on the head as the receiver. Superparamagnetic iron oxide (SPIO; Resovist, Schering, Berlin, Germany) was intravenously administered at 15 mg Fe/kg as the contrast agent to acquire steady-state CBV-weighted fMR images, using previously described settings (Shih et al., 2009, 2012; Chen et al., 2013). Enhanced neural activity increases the influx of SPIO nanoparticles, which leads to a lower signal intensity and represents an increase in regional CBV (Mandeville et al., 2004). A time-series of one hundred gradient-echo images in axial-view were obtained for CBV-weighted fMRI using the following parameters: repetition time, 150 ms; echo time, 15 ms; flip angle, 22.5°; field of view, 2.56 × 2.56 cm; slice thickness, 1.5 mm; acquisition matrix, 128 × 64 (zero-filled to 128 × 128); temporal resolution, 16 s. A total of 100 time-series images were separated equally into five phases (20 fram es for each phase) corresponding to the “off,” ‘on,’ “off,” “on,” and “off” phases of nociceptive electrical stimulation; the electrical stimulation paradigm is described below.

**Figure 3:**
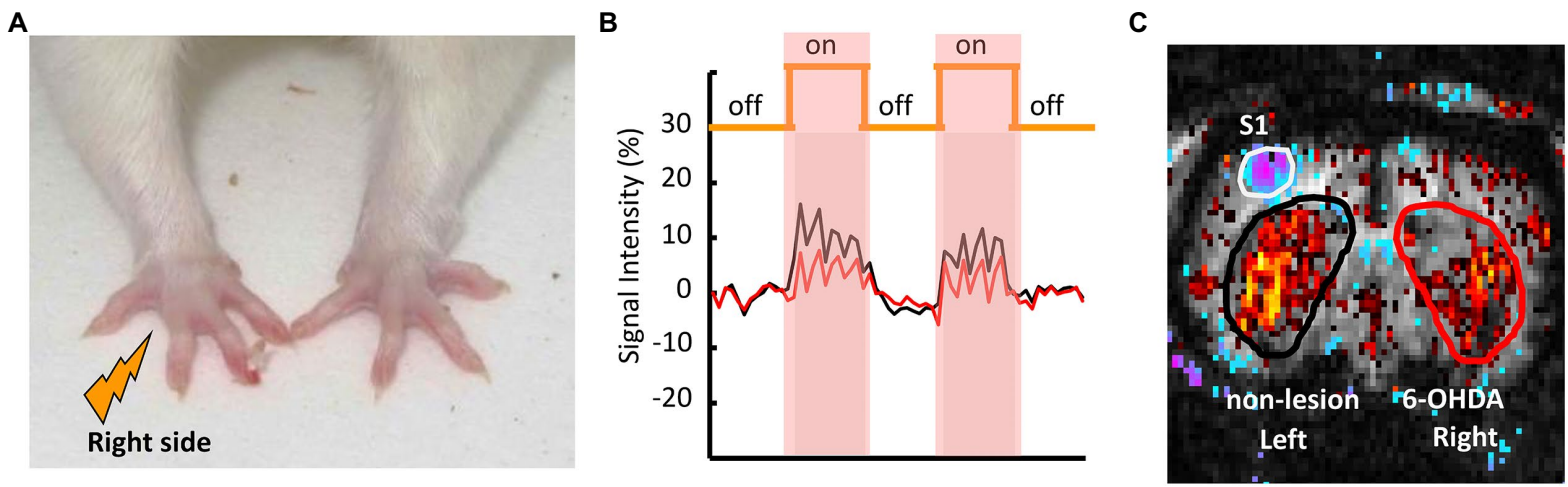
Representative fMRI scan of a PD rat and the positions of the ROIs in the 6-OHDA-lesioned and non-lesioned hemispheres. **(A)** Nociceptive electrical stimulation was applied to the right forepaw of the PD rat *via* a pair of needle electrodes. **(B)** The time course of CBV signal intensities during nociceptive stimulation in the left (intact: black) and right (lesioned: red) striata of the PD rat. **(C)** The ROIs for the left non-lesioned striatum are shown in black, and in red for the right 6-OHDA-lesioned striatum.

As shown in Figure 3A, a pair of needle electrodes were used to deliver nociceptive electrical stimulation (10 mA intensity, 3 Hz square wave, 0.5 ms pulse) to the right forepaws of the animals using an A-M Systems model 2,100 constant-current stimulator (Carlsborg, WA, United States). Figures 3B,C presents an example of the correlation map between the CBV signals and the stimulation paradigm. Correlation maps were created by plotting the correlation coefficient (CC) for the CBV signal changes and off–on–off–on–off electrical stimulation paradigm using the cross-correlation method on a voxel-by-voxel basis (Bandettini et al., 1993; Chen et al., 2013). As the experimental conditions affect this correlation, a cut-off point of r = ± 0.2 was empirically selected; this threshold effectively identifies spatial clusters in the striatum and primary somatosensory cortex (S1).

Furthermore, to assess the therapeutic effects of rVM tissue transplantation on DA innervation of the striatum, steady-state CBV weighted fMRI was conducted after intravenous administration of the dopamine D2 receptor antagonist eticlopride (S-(−)-Eticlopride hydrochloride, E101-100MG, Sigma-Aldrich; St. Louis, United States) at a dose of 1.0 mg/kg.

#### Immunohistochemistry

At the end of the study period, 6 weeks after cell transplantation, the PD and PD-rVM groups were euthanized for IHC analysis, as described previously (Weng et al., 2017). Briefly, rats were terminally anesthetized with chloral hydrate, perfused with normal saline followed by 4% paraformaldehyde (Sigma-Aldrich), and the brains were excised, post-fixed in 4% paraformaldehyde overnight at 4°C, cryprotected by immersion in 20% sucrose in 0.1 M PBS for 2 days followed by 30% sucrose in 0.1 M PBS for 2 days, and 30-μm-thick coronal sections were obtained using a Leica CM 3050 Cryostat Microtome (Leica Microsystems, Wetzlar, Germany). The sections were rinsed in phosphate-buffered saline (PBS), incubated with 1% hydrogen peroxide (Calbiochem, Torrey Pines, CA, United States) in PBS for 30 min, placed in blocking solution prevents non-specific binding of antibodies to tissue (0.5% Triton X-100 and 3% normal goat serum [Vector, Burlingame, CA, United States] in PBS), and incubated with a primary rabbit recombinant monoclonal anti-TH antibody (1:2,000; Millipore Corporation, Billerica, MA, United States; 4°C overnight), followed by secondary goat anti-rabbit biotinylated IgG antibody (1:200; Vector; 1 h), then avidin-biotin complex (1:200; Vectastain ABC kit, Vector; 60 min), developed in 3,3-diaminobenzidine (0.05%; Sigma-Aldrich) for 6 min, washed thrice with PBS, and mounted on gelatin-coated slides.

To access the number of TH-ir cells, 30 μm striatal micrographs were taken with color CCD camera attached to the confocal microscopy [OPTIPHOT-2 (10×), MICROPHOT-FXA (100× and 200×), Nikon, Tokyo, Japan or Zeiss LSM 880 confocal microscope; ZEISS]. Graft areas were measured by ImageJ, and the density of TH-ir within was expressed as the cell number/mm^3^.

For semi-quantitative measurement of TH-ir signals, 10× or 100×/200× images in the target and the reference regions (corpus callosum) were taken under the OPTIPHOT-2 microscope or MICROPHOT-FXA microscope (Nikon, Tokyo, Japan), respectively, and converted to 8-bit grayscale images (0–255 gray levels; Mausset-Bonnefont et al., 2003). The optical density (OD) of TH immunoreactivity were semi-quantitively scored using Image-Pro Plus v. 6.0 (Media Cybernetics, Inc., Bethesda, MD, United States), as follows:

OD ratio = (OD of target region − OD of corpus callosum)/OD of corpus callosum.

### Statistical analysis

Data are expressed as mean ± standard deviation. Statistical significances were analyzed using the Student’s *t*-test; *p < 0.05 was considered significant.

## Results

### Transplantation of rVM tissue improves motor deficits in the parkinsonian rats

**Figure 4:**
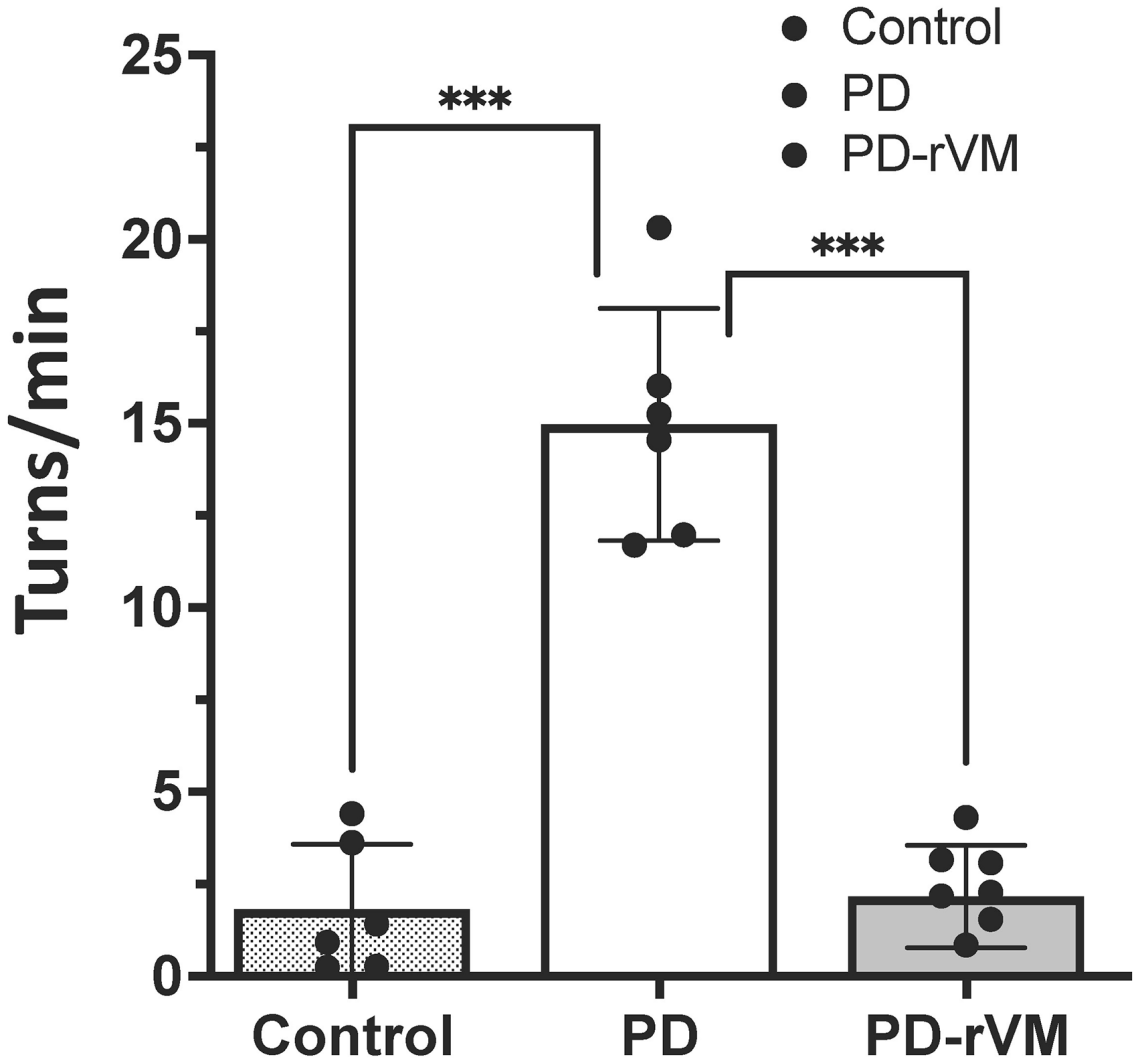
METH-induced rotation in 6-OHDA lesioned PD rats. Average turning rates of the control, PD, and PD-rVM groups. Student’s *t*-test, *n* = 6 per group; ***p < 0.005 compared to control or PD groups.

The METH-induced rotation test was carried out to evaluate the effects of cell transplantation on DA function in the parkinsonian rats. As shown in Figure 4, a significant increase in net rotation was noted in the parkinsonian rats (14.61 ± 4.89 turns/min, *n* = 6) compared to the normal control group (*n* = 6). However, after rVM transplantation, the PD-rVM group (*n* = 6) exhibited a significant improvement in motor function (2.38 ± 1.20 turns/min, ***p < 0.005 compared to the PD group).

### Transplantation relieves pain hypersensitivity in parkinsonian rats

**Figure 5:**
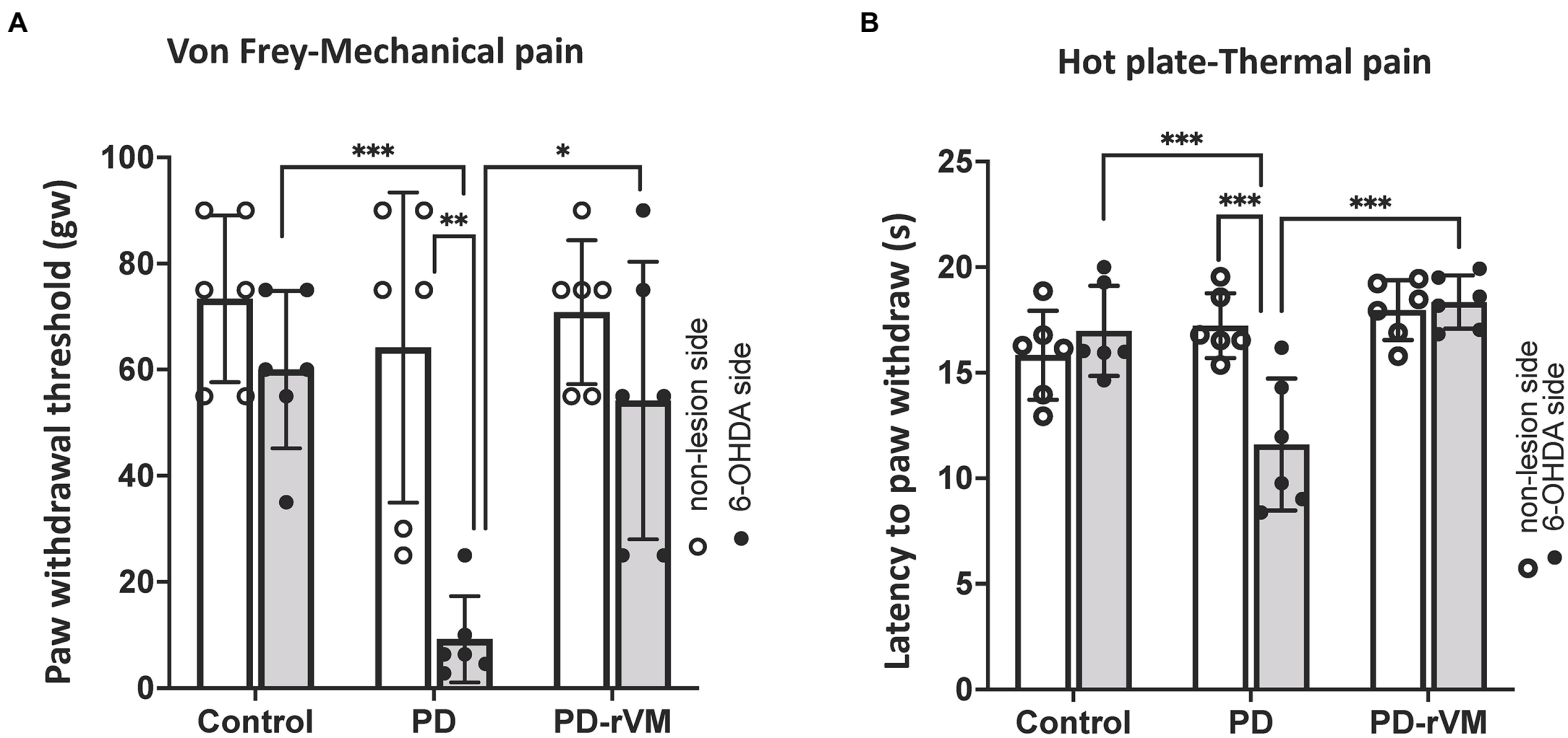
Mechanical and thermal pain tests. **(A)** In the von Frey filament test, the average paw withdrawal thresholds were significantly lower on the 6-OHDA lesioned side than the unlesioned side in the PD rats, whereas the PD-rVM group exhibited higher paw withdrawal thresholds to mechanical pain (*p < 0.05, **p < 0.01, ***p < 0.005). **(B)** In the hot plate test, the 6-OHDA-lesioned side of the PD-rVM group showed significantly longer latencies to paw withdraw compared to that of the PD group (***p < 0.005).

The paw withdrawal threshold (PWT) in response to the punctate mechanical stimuli in the Von Frey filament test was measured. In the PD group, a significant lower paw withdrawal threshold was found in the 6-OHDA-lesioned side as compared to that in the unlesioned side (**p < 0.01) or to the control group (***p < 0.005; Figure 5A). There were no significant differences in the paw withdrawal threshold between the 6-OHDA-lesioned side and unlesioned side in the PD-rVM group; however, the paw withdrawal threshold was significantly higher for the 6-OHDA-lesioned side in the PD-rVM group (*p < 0.05) compared to the ipsilesional side in the PD group (Figure 5A).

The response to the thermal stimuli was measured as the latency to paw withdrawal in the hot plate test. In PD group, the latency was much shorter in the 6-OHDA-lesioned side compared to the unlesioned side (***p < 0.005) or to the that of control group (***p < 0.005). In addition, significantly longer paw withdrawal latencies on the 6-OHDA-lesioned side were revealed in the PD-rVM group when compared to the PD group (***p < 0.005, Figure 5B).

### Transplantation of rVM tissue reduces the difference in CBV before and after nociceptive electrical stimulation in parkinsonian rats

**Figure 6:**
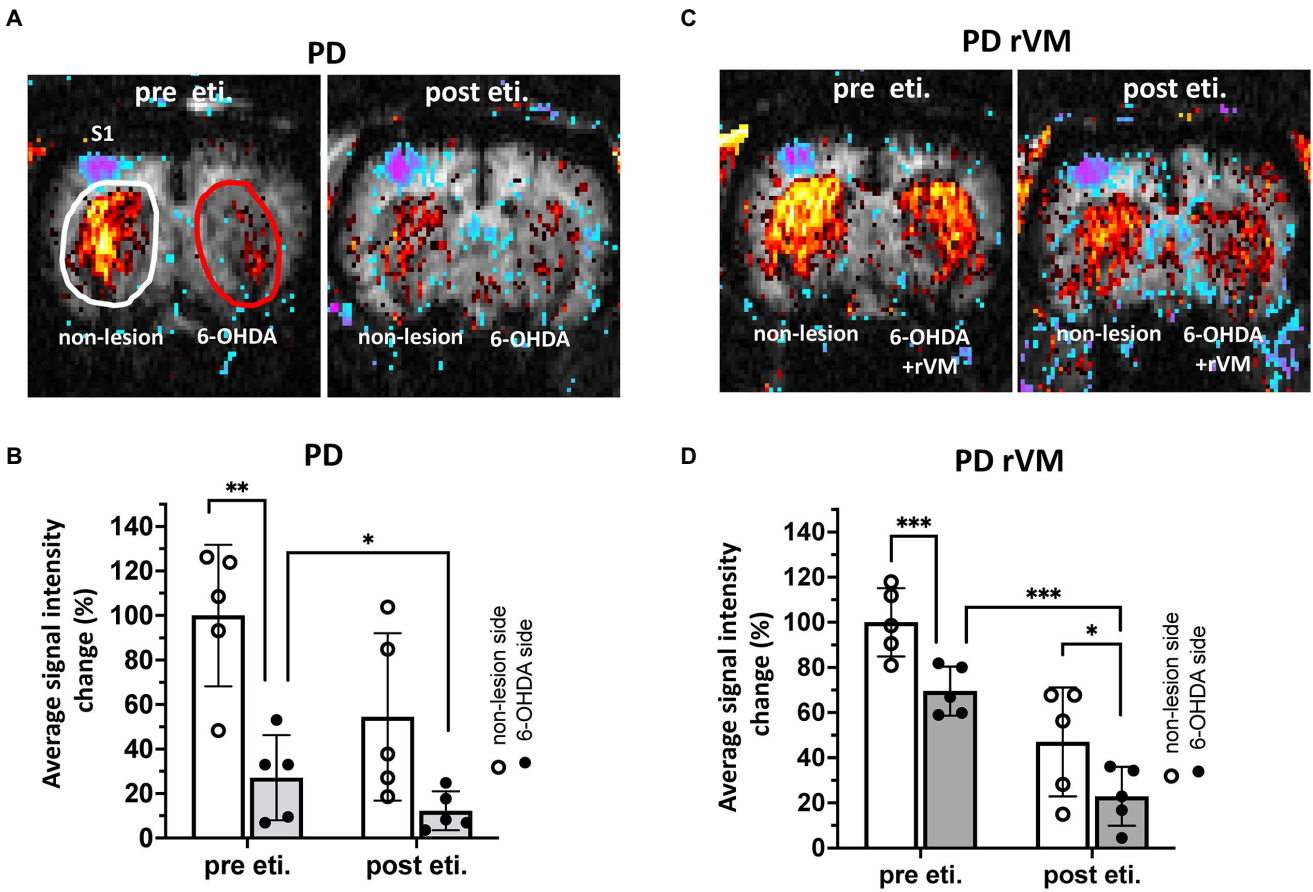
*In vivo* fMRI CBV signals in the striatum of 6-OHDA-lesioned PD rats. **(A–C)** The blue-purple signal in left primary somatosensory cortex (S1) where indicated as successful nociceptive electrical stimulation from right forepaw. Representative images of CBV signals was shown in the regions of interest, as white oval denotes non-lesion (left) side and red oval denotes the 6-OHDA side of the striatum. The quantitative results of *in vivo* fMRI CBV signals in 6-OHDA-lesioned PD rats. **(B–D)** Pre eticlopride: In the PD group, the CBV signal on the 6-OHDA-lesioned side was significantly reduced compared to the unlesioned side; however, cell transplantation in the PD-rVM group reduced this difference between the 6-OHDA-lesioned and unlesioned sides from 73 to 30%. **(B–D)** Post eticlopride: Eticlopride significantly reduced the CBV signals in both the PD and PD-rVM groups. *p < 0.05, **p < 0.01 and ***p < 0.005.

Nociceptive electrical stimulation at 3 Hz was applied to the right forepaw during fMRI imaging, and the signal, shown as blue-purple color in left primary somatosensory cortex (S1) was taken as the indication of a successful stimulation. Before applying eticlopride, the dopamine D2R antagonist, the CBV signals in the unlesioned side in PD group were significant higher in the striatum where indicated as red color when compared to the 6-OHDA lesioned side (**p < 0.01, Figures 6A,B-pre eti.).

In the group receiving cell transplantation (PD-rVM), stronger CBV signal was still found in the unlesioned side, as compared to that in the 6-OHDA-lesioned side (***p < 0.005, Figures 6C,D-pre sti.); however, the difference of blood volume between unlesioned and 6-OHDA-lesioned side was significant lower in the PD-rVM group than that in the PD group (30.5 ± 5.2% vs. 72.9% ± 23.7%, respectively, *p < 0.05, Table 1).

**Table 1:** Results of quantitative CBV analysis.

	PD				PD rVM				
	Non-lesion	6-OHDA side	*p* vs. non-lesion side	Difference between two sides	Non-lesion	6-OHDA side	*p* vs. non-lesion side	Difference between two sides	*p* vs. 6-OHDA side in PD
Pre Eti.	100.0 ± 31.8	27.1 ± 19.1	**	72.9 ± 23.7	100.0 ± 15.1	69.5 ± 10.9	***	30.5 ± 5.2	*
Post Eti.	54.4 ± 37.7	12.2 ± 8.8	*	42.2 ± 30.8	47.0 ± 24.1	22.9 ± 13.1	*	24.1 ± 14.8	***
Pre versus Post eti	##	#			##	###			

Data presents as mean ± SD. *p or #*p* < 0.05, **p or ##*p* < 0.01, and ***p or ###*p* < 0.005.

### A dopamine D2R antagonist inhibits CBV signals on the 6-OHDA-lesioned side in PD-rVM rats

Next, we used the dopamine D2R antagonist eticlopride to assess whether rVM cell transplantation restores DA function. In the PD group, administration of eticlopride largely inhibited the CBV signals in the 6-OHDA side (*p < 0.05, upper-right panel in Figures 6A,B-post eti.). Similar patterns were also observed in the PD-rVM group, as eticlopride significantly inhibited the CBV signals in the 6-OHDA-lesioned side (*p < 0.05, lower right panel in Figures 6C,D-post eti).

Moreover, there were significant differences in the CBV in the 6-OHDA-lesioned side between the PD and PD-rVM groups after administration of eticlopride (***p < 0.005, Table 1).

### Cell transplantation increases the TH-positive immunoreactivity and cells in hemi-parkinsonian rats

**Figure 7:**
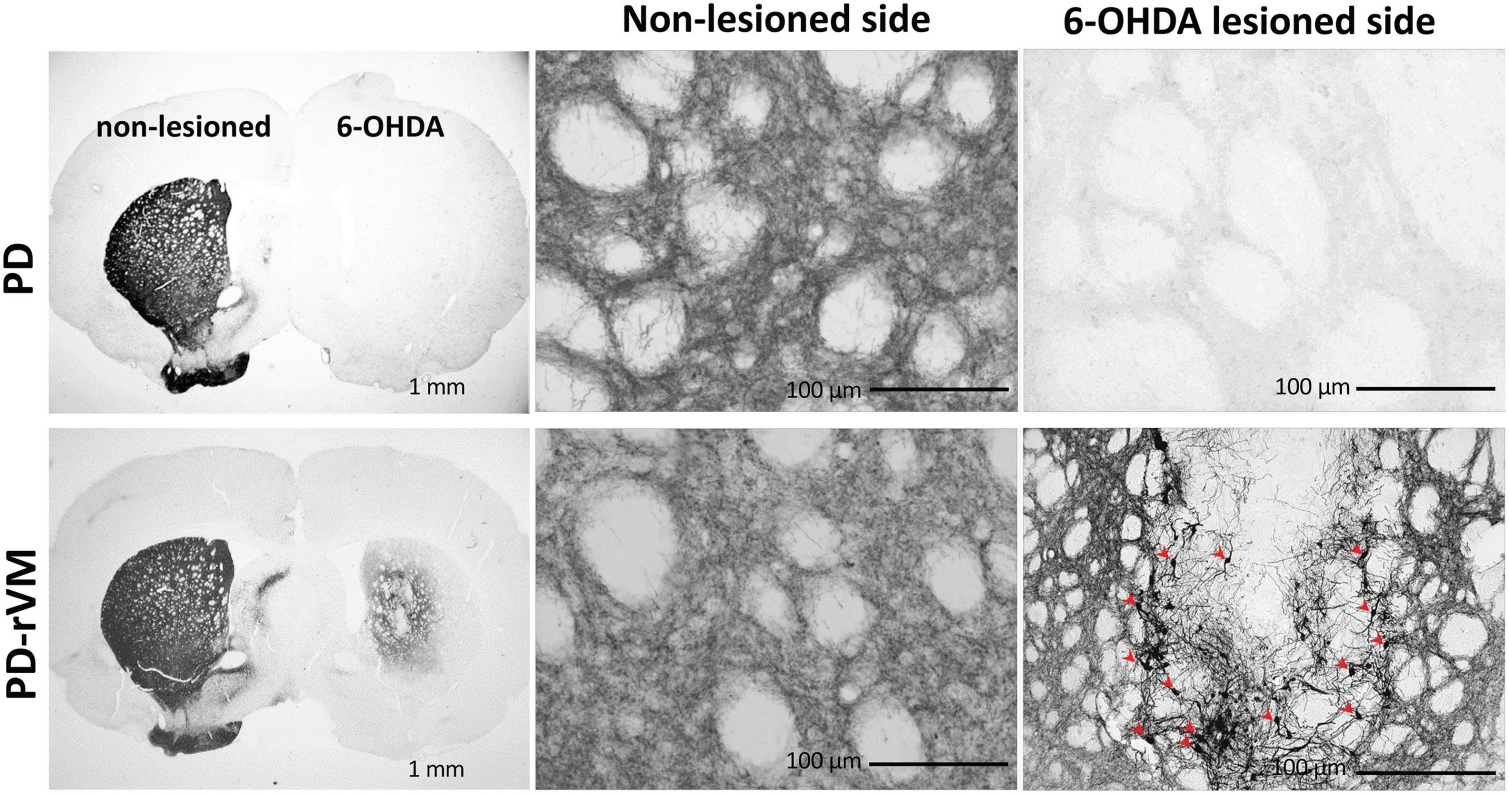
Tyrosine hydroxylase immunostaining in the brain of PD rats. In the PD group, the 6-OHDA-lesioned side exhibited much lower TH immunoreactivity than the unlesioned side. In contrast, cell transplantation significantly increased TH immunoreactivity in the PD-rVM group. The TH-positive cells in the striatum on the 6-OHDA-lesioned side of from the PD-rVM group are indicated by red arrows (lower right panel).

TH staining revealed a low density of residual fibers in the 6-OHDA lesioned striata compared to the unlesioned striata of PD rats (Figure 7, upper panels). TH immunoreactivity in the 6-OHDA-lesioned side was significantly enhanced in the cell transplantation group (PD-rVM; Figure 7, lower panels). Also, stronger TH-positive cell bodies and nerve fibers were found in 6-OHDA lesioned side in striatum of PD-rVM rats than that in PD group (red arrows in the lower right panel of Figure 7).

**Figure 8:**
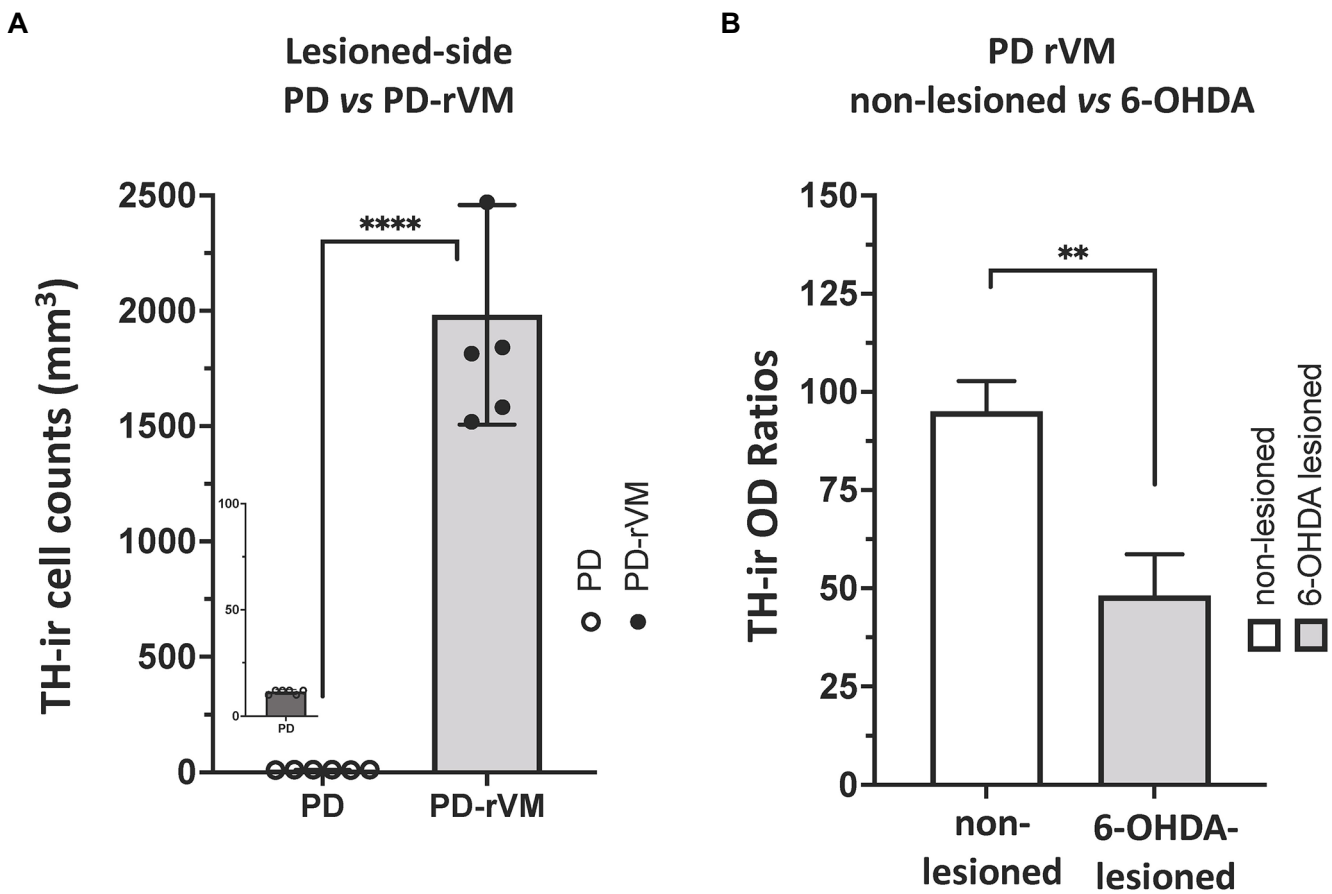
Quantitative immunohistochemical analysis of TH immunoreactivity in the striatum of PD rats. **(A)** The number of TH-positive cells was much higher in the lesioned striata of the PD rats receiving cell transplantation (PD-rVM). **(B)** In PD-rVM group, at 4 weeks after cell transplantation, TH-ir OD ratios reached to 47% when normalized to unlesioned side (100%). **p < 0.01, ****p < 0.001.

Furthermore, after cell transplantation, we also found that the number of TH-immunoreactive striatal cells was significantly higher on the lesioned side of PD-rVM group compared to that in the PD group (****p < 0.0001, Figure 8A). Semi-quantitative immunohistochemical analysis was performed by normalizing the optical density (OD ratio) of the lesioned side to that of the unlesioned side (100%). Four weeks after cell transplantation, the OD values for the 6-OHDA lesioned side were 47% in the PD-rVM rats when normalized to unlesioned side (**p < 0.01, Figure 8B).

## Discussion

In the present study, we used motor and non-motor behavior tests, *in vivo* fMRI, and immunohistochemistry to examine the anti-nociceptive effects of rVM cell transplantation in the striatum following unilateral induction of lesions in DA neurons by micro-injure of 6-OHDA into the right MFB. To the best of our knowledge, this is the first evidence of the anti-nociceptive effects of transplantation of E14 fetal rVM cells based on motor and non-motor behavior tests and *in vivo* fMRI.

We demonstrated that rats with unilateral intrastriatal 6-OHDA-induced PD exhibited profound asymmetry in motor performance tests, characterized by asymmetric body posture, impaired use of the contralateral forelimb, and sensorimotor orientation defects, i.e., loss of orientating movements elicited by application of sensory stimuli to the body contralateral to the lesion. Moreover, previous reports support the turning rates of the rats with 6-OHDA lesions in the striatum or MFB, as non-linear relationships were observed between turning and the reduction in the DA concentration in the lesioned striatum, loss of tyrosine hydroxylase (TH)-positive neurons in the substantia nigra, and loss of TH-positive innervation in the ipsilateral striatum. These results suggest that at least 40–50% reduction of the nigral TH-positive neurons, striatal TH-positive fibers, and striatal DA levels are required to invoke significant turning (Lee et al., 1996; Kirik et al., 2001; Heuer et al., 2012; Tronci et al., 2012). AMPH-induced rotation was also reported as a sensitive practical and convenient test of functional recovery in cell transplantation, as transplants containing fewer than 500 surviving DA neurons matched well with the number of surviving DA neurons and striatal DA concentration (Brundin et al., 1988; Kirik et al., 2001).

Albeit the usefulness of 6-OHDA-induced PD model was demonstrated by the METH-induced motor function tests in the evidence above and in the current study (Jagmag et al., 2015; Gomez-Paz et al., 2018), quantification analysis of TH-ir striatal and nigral cells, as well as additional motor behavior studies such as gait analysis and forelimb akinesia, provide stronger assessment of the extent of neurotoxin, i.e., MPTP or 6-OHDA in this study, elicited lesions of nigrostriatl DA neurons.

Additionally, we revealed reduced mechanical and thermal detection thresholds in the unilateral hindpaws of the unilateral 6-OHDA-lesioned rats. We also demonstrated the anti-nociceptive effects, i.e., relief of mechanical and thermal pain sensation, of rVM transplantation, which were supported by earlier studies using fetal rVM (Takeda et al., 2014) or chromospheres (Gomez-Paz et al., 2018). However unilateral SNc lesions of DA neurons impaired motor function on the contralateral side (Yoon et al., 2014; Zhou et al., 2019). This finding might partially support the notion that 6-OHDA-induced lesions result in pain hypersensitivity and motor deficits *via* different pathophysiological mechanisms. These observations may also explain why treatments for motor deficits do not produce comparable benefits for pain symptoms in patients with PD.

Noxious electrical somatosensory stimulation reduced bilateral CBV in the caudate putamen (CPu), which is associated with increased neural activity in the region involved in strong vasoactive neurotransmission (Shih et al., 2009). Furthermore, the decreases in striatal CBV modulated by D2-like dopamine receptors (D2R) observed by fMRI represent an important mechanism that occurs during neurovascular processing of nociception (Hagelberg et al., 2004; Martikainen et al., 2005). Moreover, Hsu et al. (2014) reported that changes in negative BOLD fMRI signals during nociceptive-stimulation in PD rats were associated with altered Ca2+ signaling in the DA system (Hsu et al., 2014).

Lesioning striatal DA neurons increases pain sensitivity (i.e., leads to a lower pain threshold); thus, conversely, recovery of the lesioned neurons may inhibit pain responses (Lin et al., 1984). Therefore, the present results demonstrate that the reduced CBV signals of the lesioned striatum in response to electrical stimulation could be partially explained by DA dysfunction during activation of the nigrostriatal system. Also, administration of the D2R antagonist eticlopride clearly blocked the CBV signals on the unlesioned side. These findings support the suggestion that D2R mediated decreases in CBV play a key role in the anti-nociceptive effects of pain modulation.

Additional to the recovery of behavioral function, our results also showed restored CBV signals in the rVM group, which may be explained by the re-innervation of exogenous rVM cells to dopaminergic fibers (Wakeman et al., 2011). The reduced metabolic demand or loss of cholinergic neurons innervating cortex and microvessels resulted in the reduction of CBV in PD (Globus et al., 1985). Significant region-specific decrease of CBV in the substantia nigra, caudate and putamen in PD patients compared to controls has been evidenced *via* advanced *in vivo* MR or single-photon emission computed tomography (SPECT) imaging (Paez et al., 2020) and the change of regional CBV could be considered as a clinical feature for objective evaluation of disease progression (Taguchi et al., 2019). For rVM-based therapies, there are two major factors for evaluation, i.e., efficiency of exogenous rVM cell transplantation and boosting self-repair of endogenous neural stem cells. In summary, based on our current results, we suggested rVM-based therapies could improve the CBV signal and *in vivo* monitored by using fMRI.

It should be noted that the majority of preclinical validation studies used to assess the therapeutic approaches have been performed in the 6-OHDA rodent model; however, the limitation of 6-OHDA model of PD could be while this is a useful rodent model to assess rVM therapeutic efficiency, it may not reflect the pathological features or progressive status of the disease, such as the abnormal accumulation of misfolded alpha-synuclein (a-Syn) aggregates in different brain areas and its association with neuronal degeneration in PD. Given the fact that Alpha-synuclein (a-Syn) is central to the pathology of PD, mature a-Syn based animal PD models would be valuable to investigate the disease mechanism and therapeutic strategies (Gomez-Benito et al., 2020). Transplantation of human pluripotent stem cells (hPSCs)-derived DA neurons have been reported recently (Chen et al., 2016; Nolbrant et al., 2017). However, controversies do exist surrounding the advantages and disadvantages of different tissue origins for the DA cell replacement therapy in PD. The nature of fetal VM containing high percentage of dopaminergic precursor cells may bypass cell culture or *in vitro* differentiation before transplantation (Laguna and Barker, 2009). Moreover, it appeared that no tumor formation was reported from fetal VM tissue in PD transplantation therapy (Nanni et al., 2007; Laguna and Barker, 2009). However, the access to human fetal tissue from legally terminated embryos and the difficulty to optimize, and standardize the protocols limited the application of fetal VM tissue-based therapy (Barker and Consortium, 2019; Depierreux et al., 2021).

Nevertheless, with the experiences we established, the future work would be to evaluate the potential of *in vivo* imaging tool with rVM-derived DA neurons in a-Syn-based PD model. Based on the specificity and sensitivity for the DA synthesis in the nigrostriatal pathway, PET molecular imaging of striatal [^18^F]-DOPA uptake has been demonstrated as one of the most reliable tool for the *in vivo* diagnosis of PD, as well as the measurement of terminal loss of dopamine (Nanni et al., 2007; Depierreux et al., 2021). Therefore, [^18^F]-DOPA PET and CBV fMRI coupling imaging could be a promising *in vivo* therapeutic diagnosis tool for hPSCs or D2R target therapy.

The current results showed that unilateral 6-OHDA lesions of the MFB in rats can be used to model specific changes in pain behavior with Von Frey and hot plate tests in PD. By using the contralateral side to control for individual differences and analyzing the CBV with fMRI, it is possible to assess disease progression as a function of changes in anti-nociceptive effects. Chen C V. et al. demonstrated that the hypersensitivity to pain stimulus mainly occurred on the side of the body ipsilateral to the right SN lesion and the reduced responsiveness of the striatal CBV reactions occurred ipsilateral to the right SN lesion (Chen et al., 2013). However, the interpretation of the current results was limited by the lack of sham control, and it would be required to avoid misleading or to have a better understanding between disease status of PD and normal control.

Overall, our data indicate that cell replacement therapy alleviated pain and thermal dysesthesia, and correlated with improved motor function in the PD rats. The fMRI data revealed that the grafted striatum exhibited a recovery of the CBV response to nociceptive stimuli. Moreover, we showed the recovery of the CBV signals in the grafted striatum were inhibited by the D2R antagonist eticlopride, which confirmed that rVM tissue transplantation restored DA cells. In summary, good correlations were observed between the recovery of the nociception-induced CBV responses in the motor and non-motor behavior tests and DA innervation in the IHC studies of the grafted striatum.

## Conclusion

In summary, the present study demonstrates unilateral microinjection of 6-OHDA into the MFB in rats replicates the motor and non-motor deficits of PD. We also show that transplantation of E14 fetal rVM tissue results in anti-nociceptive effects and improves motor function. Moreover, *in vivo* fMRI revealed that the E14 fetal grafts reversed the reductions in the CBV signals observed in the 6-OHDA-lesioned striatum. *In vivo* CBV signaling confirmed the key role of D2R-mediated pain modulation. Therefore, we conclude that rVM tissue transplantation represents a promising therapeutic approach for the treatment of PD-related pain.

## Data availability statement

The raw data supporting the conclusions of this article will be made available by the authors, without undue reservation.

## Ethics statement

The animal study was reviewed and approved by the institutional animal care and use committee of the National Defense Medical Center.

## Author contributions

C-HC and K-HM: conceptualization. C-HC and S-JW: methodology and software. Y-TJ and S-JW: validation. C-HC and SH-H: formal analysis and writing—original draft preparation. W-SH, H-FC, and C-YC: investigation. C-HC, S-JW, and Y-TJ: resources. K-HM and C-HC: data curation and funding acquisition. S-JW, K-HM, Y-TJ, and SH-H: writing—review and editing. C-HC: visualization and project administration. C-CY and K-HM: supervision. All authors contributed to the article and approved the submitted version.

## Funding

This research was funded by the Ministry of Science and Technology (MOST 109-2314-B-016-014-MY2, 108-2314-B-016-011-MY3 and 110-2314-B-016-029).

## Conflict of interest

The authors declare that the research was conducted in the absence of any commercial or financial relationships that could be construed as a potential conflict of interest.

## Publisher’s note

All claims expressed in this article are solely those of the authors and do not necessarily represent those of their affiliated organizations, or those of the publisher, the editors and the reviewers. Any product that may be evaluated in this article, or claim that may be made by its manufacturer, is not guaranteed or endorsed by the publisher.
